# Thyroid Volume in Pregnancy is Associated with Parity, Gestational Age, and Body Mass Index in an Iodine-sufficient Area

**DOI:** 10.1055/s-0043-1776028

**Published:** 2023-11-09

**Authors:** Mariana Couto Monteiro, Gabriela Assayag, Ricardo Botler, Anice Bergamin, Flávia Lúcia Conceição, Nathalie Silva de Morais, Roberto Perrota de Seixas, Tatiana Martins Benvenuto Louro Berbara, Annie Schtscherbyna, Carolina Martins Corcino, Débora Ayres Saraiva, Mário Vaisman, Patrícia Fátima Santos Teixeira

**Affiliations:** 1 Endocrinology Postgraduate Program, Medical School, Universidade Federal do Rio de Janeiro, Rio de Janeiro, RJ, Brazil; 2 Radiology Division, Hospital Municipal Rocha Maia, Rio de Janeiro, RJ, Brazil

**Keywords:** thyroid volume, pregnancy, iodine status, thyroid nodule, body mass index, volume tireoidiano, gestação, status iódico, nódulo tireoidiano, índice de massa corporal

## Abstract

**Objective** We compared thyroid volume (TV) and presence of nodular goiter (NG) in pregnant vs. non-pregnant women in an iodine-sufficient area. We also evaluated the relationship between gestational age, parity, and TV in the pregnant women group, and determined the 2.5 ^th^ and 97.5 ^th^ percentiles of normal TV in pregnancy.

**Methods** This cross-sectional study included 299 healthy women (216 pregnant) without previous thyroid diseases. Thyroid ultrasounds were performed and compared between pregnant and non-pregnant women. The range of normal distribution of TV (2.5 ^th^ and 97.5 ^th^ percentiles) in pregnancy was determined after excluding individuals with positive thyroid antibodies, NG, and/or abnormal serum thyrotropin (TSH) or free thyroxine (FT4).

**Results** Thyroid volume was larger among pregnant compared to non-pregnant women (8.6 vs 6.1 cm ^3^ ; *p* < 0.001) and was positively correlated with gestational age (rs = 0.221; *p* = 0.001), body mass index (BMI, rs 0.165; *p* = 0.002), and FT4 levels (rs 0.118 *p* = 0.021). Nodular goiter frequency did not differ between the two groups. There was a negative correlation between TV and TSH (rs -0.13; *p* = 0.014). Thyroid volume was lower among primiparous compared to multiparous patients (7.8 vs 8.9; *p* < 0.001) and was positively correlated with parity (rs 0.161; *p* = 0.016). The 2.5 ^th^ and 97.5 ^th^ percentiles of TV were 4.23 and 16.47 cm ^3^ , respectively.

**Conclusion** Thyroid volume was higher in pregnant compared to non-pregnant women and was positively related to parity, BMI, and gestational age in a normal iodine status population. Pregnancy did not interfere with the development of NG.

## Resumo

**Objetivo** Comparamos o volume tireoidiano (VT) e a presença de bócio nodular (BN) em mulheres grávidas e não grávidas em uma área suficiente em iodo. Também avaliamos a relação entre idade gestacional, paridade e VT no grupo de gestantes e determinamos os percentis 2,5 e 97,5 de VT normal na gestação.

**Métodos** Este estudo transversal incluiu 299 mulheres saudáveis (216 grávidas) sem doenças tireoidianas prévias. Ultrassonografias de tireoide foram realizadas e comparadas entre mulheres grávidas e não grávidas. A faixa de distribuição normal de VT (percentis 2,5 e 97,5) na gestação foi determinada após a exclusão de indivíduos com anticorpos tireoidianos positivos, BN e/ou tireotropina sérica (TSH) ou tiroxina livre (T4L) anormais.

**Resultados** O VT foi maior entre as gestantes em comparação com as mulheres não grávidas (8,6 vs 6,1 cm3; *p* < 0,001) e foi positivamente correlacionado com a idade gestacional (rs = 0,221; *p* = 0,001), índice de massa corporal (IMC, rs 0,165; *p* = 0,002) e níveis de T4L (rs 0,118 *p* = 0,021). A frequência de BN não diferiu entre os dois grupos. Houve correlação negativa entre VT e TSH (rs -0,13; *p* = 0,014). O VT foi menor entre as primíparas em comparação com as multíparas (7,8 vs 8,9; *p* < 0,001) e foi positivamente correlacionado com a paridade (rs 0,161; *p* = 0,016). Os percentis 2,5 e 97,5 de VT foram 4,23 e 16,47 cm3, respectivamente.

**Conclusão** O VT foi maior em gestantes em comparação com mulheres não grávidas e foi positivamente relacionado à paridade, IMC e idade gestacional em uma população com status iódico normal. A gravidez não interferiu no desenvolvimento de BN.

## Introduction

Pregnancy leads to important changes in thyroid physiology, with a higher demand to produce thyroid hormones. 1 2 3 4 5 During pregnancy, higher levels of estrogen increase circulating thyroxine binding globulin levels and decrease thyroid hormones free fractions, which stimulates the hypothalamic-pituitary-thyroid axis. Besides, the placental alfa subunit of human chorionic gonadotropin directly stimulates the thyroid-stimulating hormone (TSH) receptor, increasing thyroid hormone production and thyroid volume (TV). An enlargement of thyroid gland may be associated with physiological thyrotoxicosis during pregnancy due to this excessive stimulus to the gland. 2 4

The higher demand of thyroid hormone production is fulfilled when there is enough amount of iodine and typical thyroid parenchyma, especially in the absence of autoimmune diseases. 1 3 However, iodine deficiency or excess my compromise the adaptive mechanisms in maternal thyroid function. Pregnancies in conditions of iodine deficiency induce even larger volumes and goitrogenic effects since iodine deficiency is a classical goitrogen factor. In iodine-deficient areas, an increase of 20-30% of TV is reported in pregnant women, compared to non-pregnant women. 1 6

Other elements such as parity, age, serum TSH levels, and genetic characteristics seem to interfere in maternal TV. 7 However, most studies that evaluated the correlation of these variables with TV were conducted in areas with inadequate iodine intake, which could be a bias.

In this context, we proposed the present study to compare the TV and the presence of nodular goiter (NG) between pregnant and non-pregnant women from an iodine-sufficient area. Furthermore, we aimed to evaluate the relationship between demographic parameters, gestational age, number of previous pregnancies and TV in pregnant women. Finally, we determined the 2.5 ^th^ and 97.5 ^th^ percentiles of TV in pregnant women with normal thyroid function and absence of thyroid autoimmune disease, proposing a reference range for TV in women along the different trimesters of pregnancy.

## Methods

This was a sectional study enrolling 216 pregnant women aged 18 to 35 years old, without a previous history of thyroid diseases, who were attending obstetric outpatient appointments at four public health basic care units in Rio de Janeiro. All health care units were in urban areas of the state. The inclusion period was from May 2014 to January 2017. The study was approved by the local Research Ethics Committee, and all subjects signed consent forms (CAAE **:** 22546213.0.0000.5275).

Women with any chronic disease, body mass index (BMI) > 40, or any newly diagnosed disease at the first obstetric evaluation were excluded. Patients with multifetal pregnancies or with history of levothyroxine or antithyroid drug use were also excluded. An additional exclusion criterion was the use of drugs or supplements containing iodine.

In order to determine the range of normal distribution of TV in pregnancy, we excluded pregnant women with positive serum thyroid peroxidase antibody (TPOAb) and/ or thyroglobulin antibody (TgAb), NG, and abnormal serum TSH or FT4. Normal TSH reference range was defined according to the American Thyroid Association recommendations. 8

Body mass index (BMI) was calculated as weight (Kg) divided by height squared (m ^2^ ). Participants were classified as obese according to BMI classification tables specific to pregnant women. 9

As shown by Saraiva et al., the median ioduria among pregnant women in the same region was 221.0 μg/L, which reflects a sufficient iodine status according to the World Health Organization classification. 10

A control group with 83 non-pregnant women of similar age was selected. This group did not have known thyroid disease or other chronic conditions and lived in the same region. Also excluded were those who had BMI > 40 kg/m ^2^ and those taking levothyroxine or supplements containing iodine.

Serum TSH, FT4, TPOAb, and TgAb concentrations were determined by electrochemiluminescence assays with Roche Modular Analytics E170. The reference values, intra- and inter-assay variations were, respectively, TSH: 0.1 to 3.8 mIU/L 11 ; 7.2% and 3%; FT4: 0.7 to 1.9 ng/dL; 2.8% and 2.9%; TPOAb: < 34 UI/mL; 6.3% and 7.0%; TgAb: < 115 UI/mL; 4.9% and 6.3%. For non-pregnant women, serum TSH and FT4 reference ranges were 0.4 to 4.3 mUI/L and 0.7 to 1.9 ng/dL.

All participants underwent a thyroid ultrasound evaluation. Considering the pregnant group, the majority (n = 136) was evaluated in the first trimester; however, a group of 37 pregnant patients had US assessment in the second or third trimester. All thyroid ultrasound scans were performed by one of three trained examiners (NSM, PFST, RPS) using a high-frequency SIEMENS-AUSONX 300 (Siemens AG, Munich, Germany) or MYSONO U5 SAMSUNG transducer (12 MHz) (Samsung Electronics Co., Ltd., Suwon-si, South Korea). Thyroid volume was calculated as the summation of each lobe and isthmus volumes, using the formula: length x width x thickness x 0.52. 12 The presence, location and size of thyroid nodules were also evaluated and described. Nodular goiter was defined as the presence of one or more solid lesions ≥ 3.0 mm in diameter in both transverse and longitudinal axes. Thyroid nodule volume was calculated by length × width × thickness × 0.52. 12

Statistical analysis was performed using the IBM SPSS Statistics for Windows, version 24.0 (IBM Corp., Armonk, NY, USA). Continuous variables were described as median (interquartile range) and categorical variables as frequencies. Comparations were performed using the Chi-square and Fisher exact tests for categorical variables. The Mann-Whitney U test was used to compare continuous variables. A *p* -value < 0.05 was considered significant.

## Results

A total of 299 women (216 pregnant) were evaluated. They did not differ regarding age (median age = 28.0 years old in both groups *p* = 0.549) and were also comparable regarding the frequency of overweight and obesity, as depicted in Table 1 . Median serum TSH was lower in the pregnant group compared to the non-pregnant group (1.3 vs 2.0 mUI/L; *p* < 0.001) and, in contrast, median FT4 was higher (1.2 vs 1.0 mUI/L; *p* < 0.001). The frequency of NG as well as the number and volume of thyroid nodules did not differ between the two groups ( Table 1 ).

**Table 1:** Baseline characteristics of the study population, comparing pregnant and non-pregnant women

	Pregnant	Non-pregnant
Age (years)	28.0 (8.0)	28.0 (7.0)
Overweight (%)	30	33.7
Obesity (%)	16.9	27.7
TSH (mUI /L)	1.35 (1.4) a	2.01 (1.8)
FT4 (ng/dL)	1.2 (0.3) a	1.0 (1.1)
Thyroid volume (cm ^3^ )	8.6 (3.7) a	6.1 (3.2)
Nodular goiter (%)	9.7	16.9
Number of nodules (%)	0.12 (0.06–0.17)	0.28 (0.11–0.46)
Total nodular volume (cm ^3^ )	0.24 (0.32)	0.18 (0.61)

Abbreviations:

TSH, thyrotropin; FT4, free thyroxine.

a *P* -value < 0.01 compared with the non-pregnant group.

Continuous variables are presented as median (interquartile range).

Thyroid volume was larger among pregnant compared to non-pregnant women (8.6 vs 6.1 cm ^3^ ; *p* < 0.001) and was positively correlated with gestational age (rs = 0.221; *p* = 0.001), BMI (rs 0.165; *p* = 0.002) and FT4 levels (rs 0.118 *p* = 0.021). There was a negative correlation between TV and TSH (rs -0.13; *p* = 0.014). Thyroid volume was lower among women in their first trimester of pregnancy compared to those in the second or third trimesters (7.8 vs 8.9; *p* < 0.001) and was positively correlated with the number of previous pregnancies (rs 0.161; *p* = 0.016), as shown in Table 2 .

**Table 2:** Correlations between thyroid volume and studied variables in pregnant women

	r ^s^	*p* -value
Gestational age	+0.221	0.001
Parity	+0.161	0.016
TSH	-0.132	0.014
FT4	+0.118	0.021
Age	+0.124	0.160
BMI	+0.165	0.002

Abbreviations: BMI, body mass index; FT4, free thyroxine; rs, Spearman coefficient score; TSH, thyrotropin.

In order to determine the range of normal distribution of TV in pregnancy, we excluded 43 women with positive serum TPOAb and/ or TgAb, NG, and/or abnormal thyroid function. Among the remaining 173 pregnant women, the 2.5 ^th^ and 97.5 ^th^ percentiles of TV were 4.23 and 16.47 cm ^3^ , respectively. There was a tendency for a higher 95 ^th^ percentile of TV among pregnant women in the second/third trimesters compared to those in the first trimester (17.66 vs 12.63, *p* = 0.111), Table 3 .

**Table 3:** Ranges of distribution of thyroid volume among pregnant women without thyroid diseases

	All pregnant women* (n = 216)	First trimester (n = 136)	Second/third trimester (n = 37)
5 ^th^ – 95 ^th^	4.77–12.66	4.88–12.63 ^a^	4.19–17.66 ^a^

*Excluded patients with positive serum thyroid peroxidase antibody and/or thyroglobulin antibody, nodular goiter, and abnormal thyroid function. *P* -value = 0.111 comparing pregnant women in the first vs. second/third trimesters.

## Discussion

The results of the present study reinforce that, even in iodine-sufficient areas, pregnancy leads to a goitrogenic effect in the thyroid, also associated with higher FT4 and lower TSH levels. This goitrous stimulus persisted into the later stages of pregnancy. Also, a possible cumulative effect may exist with multiple pregnancies. In clinical practice, it may impact the approach to women with NG, since a persistent augment in TV might be expected with pregnancy.

In the studied sample of pregnant women, TV was positively correlated with parity, in consonance with previous studies demonstrating that the goitrogenic effect in the thyroid during pregnancy is not fully reversible after parturition. 12 13 14 15 16 A possible cumulative effect is supported by these results.

In accordance with some previous observations, although parity increases with age, our results indicated that there was no correlation between TV and age per se. 15 17 18 Rotondi et al. also demonstrated that the correlation between TV and the number of term pregnancies was maintained after age adjustment, highlighting parity as an independent variable correlated with TV. 15

The main difference between our study and other above-mentioned studies is that the majority were performed in iodine-deficient areas. 13 14 15 16 This condition has a goitrogenic effect on the thyroid, acting directly in the correlation of different variables and TV. 1 6 19 In this way, iodine deficiency could be an important confounding bias, particularly when considering pregnant patients.

Another study, also conducted in an iodine-sufficient area in Brazil, did not demonstrate a correlation between TV and parity. 20 The authors considered the possibility that iodine sufficiency could have a protective role against the goitrogenic effect of parity. However, they also highlighted that these findings could be due to the uniformity of the sample regarding low parity.

Our data reinforce previous evidence of an association between parity and TV, now demonstrated in an iodine-sufficient area. 10 These results bring to light the idea of an independent effect of pregnancy in the increase of TV. Despite this, we did not find differences in the frequency of NG or the number and volume of thyroid nodules among pregnant and non-pregnant women, which may be a limitation of the study design. A prospective study would be helpful to better evaluate this association.

There were other studies investigating the association between TV and BMI. Most of them suggested that TV is signiﬁcantly correlated with BMI. 20 21 One of them also demonstrated a significant decrease in TV in obese women who lost > 10% body weight. 21 Our results are in agreement with this evidence, showing a positive correlation between TV and BMI.

Finally, few previous studies have evaluated the normal distribution of TV in pregnant women. The 2.5 ^th^ and 97.5 ^th^ percentiles of TV distribution were 4.23 and 16.47 cm ^3^ , respectively. It may help future researchers when designing studies of thyroid morphology in women from the same region and may help to stablish a parameter of normality for TV in pregnancy. Moreover, as expected, there was a tendency to increase the TV values of normality in the second and third trimesters compared to the first trimester, reinforcing once again the idea of the goitrogenic effect of pregnancy.

The limitations of the present study are related to its sectional design since it would be interesting to assess TV evolution, as well as thyroid nodules development, after the delivery. Also, the small sample size should be addressed despite not being impeditive to detecting positive associations. Furthermore, we have only assessed anti-thyroid antibodies levels in the group of pregnant patients.

## Conclusion

In conclusion, we demonstrated that pregnancy has a goitrogenic effect over the thyroid, even in pregnant women living in an iodine-sufficient area. TV in pregnancy was positively related to parity, BMI, and gestational age. The found reference range for TV in pregnancy was 4.23 to 16.47 cm ^3^ .
